# Insights into the Genetic Architecture of Bran Friability and Water Retention Capacity, Two Important Traits for Whole Grain End-Use Quality in Winter Wheat

**DOI:** 10.3390/genes11080838

**Published:** 2020-07-23

**Authors:** Sviatoslav Navrotskyi, Vikas Belamkar, P. Stephen Baenziger, Devin J. Rose

**Affiliations:** 1 Department of Food Science & Technology, University of Nebraska-Lincoln, Lincoln, NE 68588, USA; snavrotskyi@huskers.unl.edu; 2 Department of Agronomy & Horticulture, University of Nebraska-Lincoln, Lincoln, NE 68583, USA; pbaenziger1@unl.edu

**Keywords:** wheat quality, wheat milling, wheat hardness, puroindolines, water absorption capacity

## Abstract

Bran friability (particle size distribution after milling) and water retention capacity (WRC) impact wheat bran functionality in whole grain milling and baking applications. The goal of this study was to identify genomic regions and underlying genes that may be responsible for these traits. The Hard Winter Wheat Association Mapping Panel, which comprised 299 lines from breeding programs in the Great Plains region of the US, was used in a genome-wide association study. Bran friability ranged from 34.5% to 65.9% (median, 51.1%) and WRC ranged from 159% to 458% (median, 331%). Two single-nucleotide polymorphisms (SNPs) on chromosome 5D were significantly associated with bran friability, accounting for 11–12% of the phenotypic variation. One of these SNPs was located within the *Puroindoline-b* gene, which is known for influencing endosperm texture. Two SNPs on chromosome 4A were tentatively associated with WRC, accounting for 4.6% and 4.4% of phenotypic variation. The favorable alleles at the SNP sites were present in only 15% (friability) and 34% (WRC) of lines, indicating a need to develop new germplasm for these whole-grain end-use quality traits. Validation of these findings in independent populations will be useful for breeding winter wheat cultivars with improved functionality for whole grain food applications.

## 1. Introduction

Numerous studies have associated the consumption of high-fiber foods with decreased risk of type 2 diabetes, obesity, and heart disease [1,2]. Consumer awareness of the health benefits of dietary fiber-rich foods has driven product development of high-fiber foods in the baking industry. As a good source of fiber, whole wheat flour has become increasingly popular globally with the number of whole grain products increasing from 164 in 2000 to 3378 in 2011 [3].

Unlike refined white flour, whole wheat flour contains the all the anatomical portions of the grain are together in the same proportions as they occur in the intact kernel [4]. The bran component (including the outer portions of the kernel: pericarp, germ, and aleurone) has a significant impact on whole wheat flour functionality [5,6,7]. Due to its high fiber concentration, wheat bran binds considerable amounts of water, which interferes with proper gluten hydration and leads to detrimental effects on bread quality [8]. Bran water retention capacity (WRC), which is a measure of how much water the bran will imbibe, varies among genotypes and growing conditions and is correlated with whole grain flour functionality [5,9]. Therefore, WRC is an important trait in the evaluation of bran quality.

As wheat is milled, the bran may fracture into many small particles or remain as larger pieces. The tendency for bran to fracture into smaller pieces is known as friability. Initial research on wheat milling was focused on a reduction in bran friability (ability to retain large particles) to facilitate efficient separation of white flour from the bran by sifting [10]. However, as whole wheat flour has become more popular, there have been numerous studies showing that bran particle size affects whole wheat flour functionality [6,11]. Reported results are somewhat contradictory, but they do show that very large particles are undesirable due to interference with gluten network formation [6]. Furthermore, bran WRC also increases with increased bran particle size [12]. Therefore, for whole wheat flour, it is desirable to have high bran friability.

In recent years, genome-wide association studies (GWAS) have been used extensively to determine the genetic basis of agronomic, physiological, and disease resistance traits in bread wheats [13,14,15,16,17,18,19,20,21,22,23,24,25,26,27,28,29,30,31,32,33]. Relatively fewer studies have used GWAS to identify loci that control end-use quality traits, including grain protein concentration, flour yield and color, gluten strength, and mixing properties, among others [16,34,35,36,37,38,39,40,41]. However, these studies have focused on end-use quality for refined wheat flour applications. No studies have focused on quality traits related to whole grain end-use quality.

Our previous study showed that among eight traits measured on wheat bran, including phenolic compounds, antioxidant activity, protein, ash, lipoxygenase activity, and free thiol groups, bran particle size and WRC showed the most significant impact on whole wheat bread volume and texture among the other chemical and physical parameters tested [9]. Therefore, the objectives of this study were to: (1) investigate the variation in bran friability and WRC in the Hard Winter Wheat Association Mapping Panel (HWWAMP), a diverse set of 299 winter wheat lines representing germplasm from the hard winter wheat growing regions of the US [42,43]; (2) conduct a genome-wide association study (GWAS) to identify genomic regions associated with bran friability and WRC; and (3) identify underlying genes associated with bran friability and WRC and investigate their annotations to determine potential genes that may be important for these two traits.

## 2. Materials and Methods

### 2.1. Plant Material 

The study comprised 299 genotypes from the HWWAMP. These genotypes included varieties released since the 1940s and represent existing germplasm of the hard winter wheat growing regions of the US, including Colorado, Kansas, Michigan, Montana, Nebraska, North Dakota, Oklahoma, South Dakota, and Texas [42,43]. The HWWAMP represents diverse germplasm with unique haplotypes as compared to a breeding program nursery from a single breeding program. A list of the genotypes comprising the HWWAMP along with their pedigrees and geographical origin has been published previously in the supplementary material of Guttieri et al. [44]. Many studies in bread wheat rely on using this HWWAMP for mapping genomic regions important for a trait, and subsequently validate those marker-trait associations in their own breeding programs prior to using the markers for marker-assisted selection. The HWWAMP has been successfully used for mapping genomic regions for various biotic and abiotic traits including wheat spot blotch resistance [45], drought resistance [46], Cd accumulation [44,47], and bacterial leaf streak resistance [48]. All genotypes in HWWAMP were grown at Lincoln, NE in 2018 in 1 m^2^ plots with fungicide application, and the harvested seeds from these genotypes were used in this study.

### 2.2. Bran Functionality Traits

Wheat kernels from each of the HWWAMP genotypes were cleaned and tempered to 15.3% moisture for 24 h. Milling was performed on a laboratory mill (Quadrumat, C.W., Jr.; Barabender Instruments Inc., South Hackensack, NJ, USA) as described previously [49]. The mill produced two fractions, bran and flour, that were collected and weighed. Bran yield was calculated as mass of bran divided by the combined mass of the flour and bran, expressed as a percentage. Bran yield was used in downstream analysis as a covariate.

Friability of bran was measured by sieving the bran fraction through two testing sieves stacked on top of each other (No. 20 and No. 60 containing 850 µm and 250 µm openings, respectively). Sieves were selected according to published guidelines, where coarse bran is separated from “shorts”, or fine bran, by sifting on a No. 20 sieve, and any remaining flour is separated from shorts by sifting through a No. 60 sieve [49]. Friability was calculated as the weight fraction of bran remaining on sieve No. 60 (fine bran particles) divided by the combined weights of bran on the No. 20 and No. 60 sieves (coarse and fine bran particles), expressed as a percentage.

The WRC of bran was analyzed according to the approved method for flour [49], with some modifications. In particular, 1 g of bran was weighed into a tared tube to which 5 mL of water was added. After vortex mixing for 5 s, samples were shaken on a horizontal shaking platform at room temperature and 100 rpm for 20 min. Then samples were centrifuged at 1000× *g* for 15 min. The supernatant was then carefully decanted and test tubes were drained upside down over paper towels for 10 min. Following decanting, the tubes were weighed and the weight of the bran plus absorbed water was calculated. WRC was calculated as the weight of bran plus absorbed water divided by the dry weight of bran, expressed as a percentage.

### 2.3. Statistical Analysis of Bran Functionality Traits

Data distributions of friability and WRC were visualized using histograms. The normality of the data was analyzed using the Shapiro–Wilk test using the “shapiro.test” package in R (version 1.2.1335, RStudio, Boston, MA, USA).

### 2.4. Genotyping and Population Structure

The HWWAMP panel was previously genotyped using the 90 K SNP wheat iSelect assay and the marker data is available at the T3 wheat database [50]. The majority of SNPs included in the 90 K SNP assay were SNPs discovered using RNA-seq transcripts from 19 hexaploid and 18 tetraploid wheat lines in addition to SNPs previously discovered in hexaploid wheat [51]. The source of majority of SNPs, being RNA-Seq transcripts, resulted in nonrandom distribution of SNPs in the genome [52,53]. Additional details on the wheat iSelect assay have been presented previously [54]. The dataset comprised a total of 16,054 SNPs across 299 genotypes. In order to avoid spurious marker–trait associations, SNP markers with minor allele frequency <0.05 and missing data of >20% were excluded from the analysis. Genotypes with >20% missing SNP sites (Dawn and Parker) were also excluded from the analyses (leaving 14,661 SNPs). The population structure of the HWWAMP was studied using principal component analysis (PCA). The PCA was performed using TASSEL (version 5.0, Buckler Lab, Ithaca, NY, USA) [55] and a biplot of PC1 versus PC2 was generated.

### 2.5. Estimating Heritability Using Genome-Wide SNP Markers

The two traits recorded in this study, bran friability and WRC, had one phenotypic value per line. This was similar to one phenotypic measurement generally available on other quality traits in a cultivar development program [56]. A single value per line did not allow the estimation of heritability using variance components derived from a linear mixed model used for phenotypic analysis. Hence, the SNP markers were used to estimate the heritability of friability and WRC traits. The quality-filtered markers used in GWAS were imputed using mean imputations and a genomic relationship matrix (GRM) was built [57]. The details of building a GRM and fitting a genomic best linear unbiased prediction (GBLUP) model to the phenotypic measurements is described previously in our earlier study [58]. The GBLUP model was fit using the Bayesian Generalized Linear Regression (BGLR) function in the BGLR package in R and variance components for GRM (genomic variance) and residual term were estimated [57]. The heritability was estimated as the ratio of genomic variance to the sum of genomic and error variances.

### 2.6. Genome-Wide Association Study (GWAS)

The GWAS was conducted in TASSEL. Several linear models were examined to achieve the best fit possible, including: general linear model (GLM) [59] using only the SNP markers as fixed effect; mixed linear model (MLM) [60] with kinship as a random effect [59]; MLM with kinship and adding principal components as covariates; and MLM with kinship and phenotypic traits (bran yield and friability) as covariates. The model fit was evaluated by visualizing QQ-plots plotted using the “qqman” package in R [61]. The model that provided the best fit was used to generate the significance values, additive effect scores, and the amount of phenotypic variation explained by the markers. Manhattan plots displaying the significance of the SNPs after Bonferroni correction for each of the traits were plotted using the “qqman” package in R.

### 2.7. Candidate Gene Analysis

The annotations of the candidate genes underlying or flanking (±5 kb) the SNPs associated with a trait were obtained from the International Wheat Genome Sequencing Consortium (IWGSC) [62]. If annotations for underlying genes were not available, then a default BLASTN search was performed using the wheat gene coding sequence (CDS) against the Ensembl Plants database of monocot and dicot sequences [63]. The BLAST search was performed to determine the likely annotation of the candidate genes. The BLAST search was limited to the top hit with an E-value cut off of less than 1 × 10^−40^ and sequence identity greater than 50%. The annotations for the underlying or flanking genes were compared against the trait of interest to determine the candidate genes that were likely important for bran functionality traits.

### 2.8. Linkage Disequilibrium between Trait-Associated SNP Markers on Chromosome 5D and Pin Locus

The Pina-D1, Pinb-D1b, and Pinb-Wild molecular marker information on 44 lines in the HWWAMP was obtained from the regional breeding programs, Northern Regional Performance Nursery (NRPN) and Southern Regional Performance Nursery (SRPN) from 2005–2009 dataset [64]. The “cor.test” procedure was conducted in R using “ggpubr” package to calculate correlation coefficients (as a measure of linkage disequilibrium (LD)) between trait associated SNP markers on chromosome 5D and Pin markers.

## 3. Results and Discussion

### 3.1. Bran Functionality Traits

With the emphasis on consumption of whole grain foods by governmental agencies [63], it is relevant to identify and develop wheat lines with specialized traits for whole grain products. Bran particle size and bran WRC have been identified as important determinants of whole grain breadmaking quality [5,8,9]. Therefore, we examined bran friability and WRC in a diverse set of 299 hard winter wheat genotypes to identify genomic regions that may be associated with whole grain end-use quality. 

**Figure 1:**
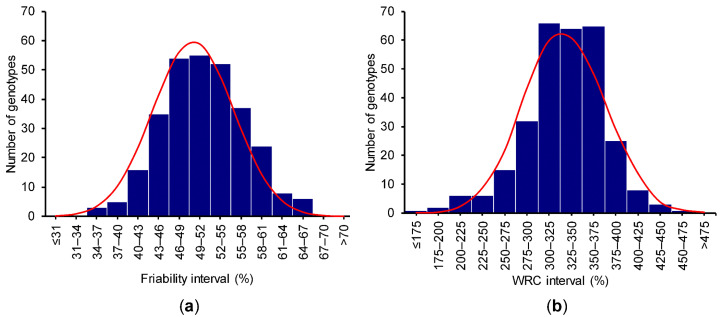
Data distribution of the selected traits: (**a**) bran friability and (**b**) water retention capacity; normal distribution curve shown in red.

Bran friability was normally distributed from 34.5% to 65.9% with a median of 51.1% (Figure 1a; Shapiro–Wilk, W = 0.99, *p* = 0.83). The wide range in this trait suggested a significant variation in milling performance among wheat genotypes in the HWWAMP.

The WRC ranged from 159–458% with a median of 331% (Figure 1b). The WRC data distribution was close to normal, but had a significant Shapiro–Wilk test (W = 0.97; *p* < 0.001). The broad range in WRC data suggested a significant genetic effect on this trait. However, unlike friability, the distribution departed slightly from normal.

Correlations among bran yield, friability, and water retention were calculated in order to determine their application as a covariates in the GWAS models. Only bran yield and WRC were significantly correlated (r = 0.21; *p* = 0.0002).

### 3.2. Genotyping and Population Structure

**Figure 2:**
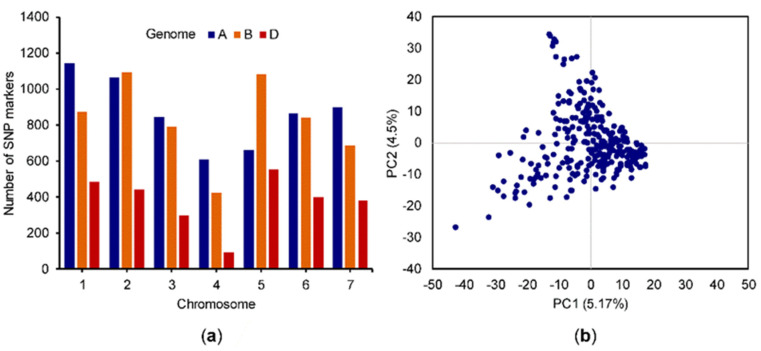
(**a**) Count of single-nucleotide polymorphisms (SNPs) on the wheat chromosomes (208 SNPs located on unanchored scaffold are not included in the plot); (**b**) principle component analysis of the 297 genotypes in the Hard Winter Wheat Association Mapping Panel (HWWAMP) using marker data.

Of the 14,731 SNPs identified across the 299 genotypes, two genotypes and 70 SNPs were dropped from analysis due to minor allele frequency <0.05 and missing data of >20%. The remaining high-quality 14,661 SNPs across 297 lines were used in the analysis. These SNP markers were distributed across all 21 chromosomes with an average of 688 SNPs per chromosome (Figure 2a). Population structure, as visualized by PCA, indicated the presence of a few outliers among the genotypes in the HWWAMP (Figure 2b). A model-based clustering performed using the program STRUCTURE has previously indicated presence of four subgroups in the HWWAMP, and the plots are available in the supplementary files of Ayana et al. [45] and Sidhu et al. [65]. The STRUCTURE plots indicated presence of just a few lines with high (>80–90%) membership to each subgroup and the rest of the lines as admixed. These results of PCA and STRUCTURE suggesting a few lines with distinct backgrounds and a large number of remaining lines as admixed is expected since many of the public wheat breeding programs routinely share germplasm among each other for use as parental lines in breeding programs.

### 3.3. Heritability Estimates for Bran Functionality Traits Using Genome-Wide SNP Markers

The genomic and error variance estimates for bran friability were 17.64 and 20.53, and for WRC they were 473.78 and 1768.09. The marker-based heritability of friability and WRC were 0.46 and 0.21. These estimates are conservative as compared to broad-sense heritability estimates obtained using the phenotypic data, because only the additive variance is captured with the markers. Friability had a moderately high heritability value, and this is in the range of heritability values recorded for most quality traits [56]. The relatively low heritability value of WRC suggests a slightly larger influence of nongenetic factors influencing these phenotypic measurements, and additional experiments will be needed to confirm the heritability for this trait. Nevertheless, since the marker-based heritability value was above zero WRC does appear to be influenced at least somewhat by genetics (the phenotypic values are not just noise or nongenetic factors), and thus can be further considered for marker–trait analysis.

### 3.4. GWAS for Bran Friability

**Figure 3:**
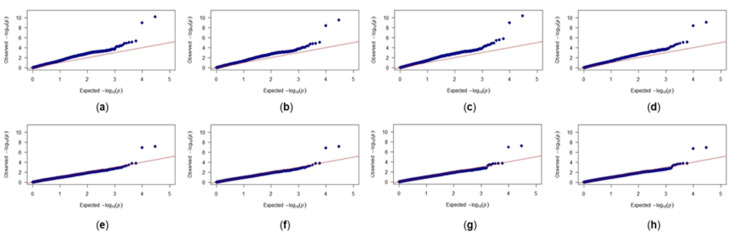
Quantile-quantile plots for bran friability trait: (**a**) General linear model (GLM) with no covariates; (**b**) GLM with bran yield (BY) as covariate; (**c**) GLM with water retention capacity (WRC) as covariate; (**d**) GLM with BY and WRC as covariates; (**e**) mixed linear model (MLM) with kinship and no covariates; (**f**) MLM with kinship and BY as covariates; (**g**) MLM with kinship and WRC as covariates; (**h**) MLM with kinship and BY and WRC as covariates. red line: X = Y; blue diamonds: actual values.

**Figure 4:**
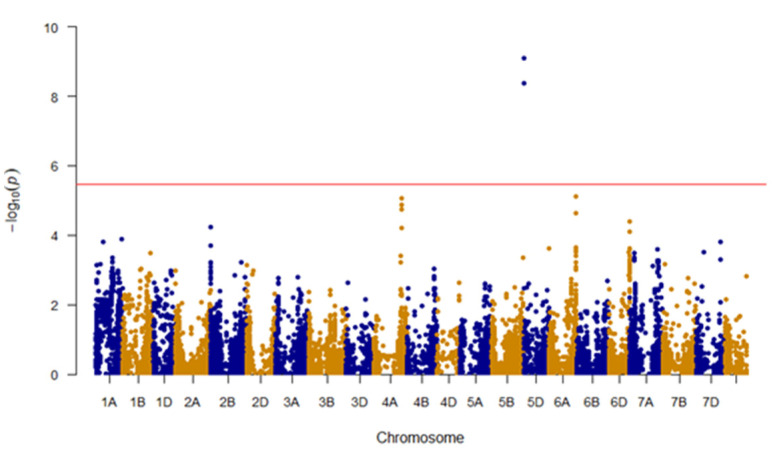
Manhattan plot showing trait associated SNPs for friability; Un, unanchored scaffold; red line, Bonferroni correction (adjusted *p* < 0.05) cutoff.

Among models tested, the MLM with kinship and bran yield as a covariate provided the best fit to the data and was used in further analysis (Figure 3). GWAS analysis identified two SNPs located on the 5D chromosome: BS00000020_51 (3,609,894 bp) and Excalibur_c49805_63 (1,614,602 bp), that were significantly associated with bran friability (Bonferroni corrected *p*-value < 0.05; Figure 4). Of the 297 genotypes, 46 carried the GG genotype (BS00000020_51 marker) and 43 carried the AA genotype (Excalibur_c49805_63 marker), which were associated with increased bran friability (Table 1; Supplementary Table S1). These genotypes were randomly distributed on a PCA biplot generated using the marker data, suggesting the absence of groupings among lines carrying the favorable alleles (Supplementary Figure S1).

Low bran friability is desirable for refined flour production because bran particles remain large and intact and are thus easier to separate from the endosperm flour. As refined flour represents the majority of wheat flour production [3], this may be why so few lines carried the high bran friability alleles. However, for whole wheat flour milling, the high bran friability trait would be beneficial because smaller bran particle sizes have been shown to result in bread with higher volume [6]. As demand for whole wheat flour increases [3], wheat genotypes that carry the high bran friability trait may become more sought after. The markers associated with bran friability in this study, after validation in independent population, will be a useful resource for developing lines with increased bran friability. The 43 to 46 genotypes carrying increased bran friability favorable alleles can be used as parental lines while making crosses to develop lines with increased bran friability (Supplementary Table S1).

### 3.5. GWAS for Bran WRC

**Figure 5:**
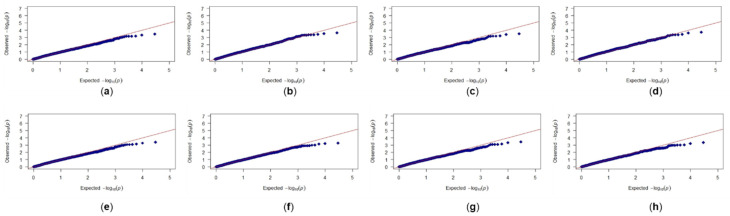
Quantile-quantile plots for water retention capacity trait: (**a**) GLM model with no covariates; (**b**) GLM model with bran yield (BY) as covariate; (**c**) GLM model with bran friability (BF) as covariate; (**d**) GLM model with BY and BF as covariates; (**e**) MLM model with kinship and no covariates; (**f**) MLM model with kinship and BY as covariate; (**g**) MLM model with kinship and BF as covariate; (**h**) MLM model with kinship and BY and BF as covariates.

**Figure 6:**
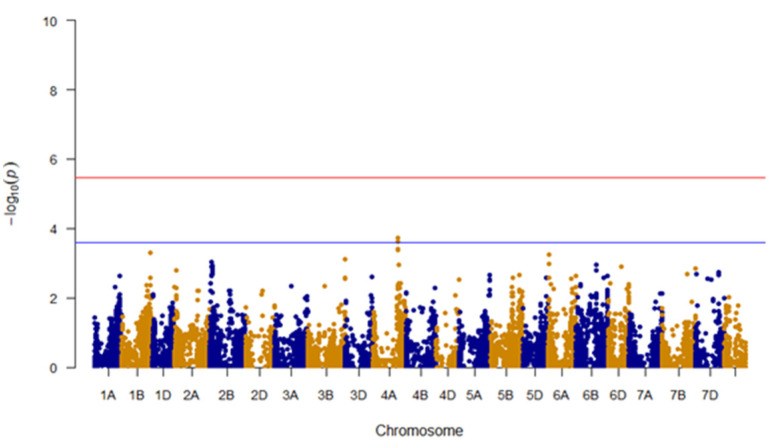
Manhattan plot showing trait associated SNPs for water retention capacity; Un, unanchored scaffold; red line, Bonferroni correction (adjusted *p* < 0.05) cutoff; blue line, notable SNPs above the background.

Among models tested, the best fit for the WRC trait was obtained using a GLM model with bran yield and friability as covariates (Figure 5). Unlike the friability trait, GWAS analysis did not reveal any makers that were significantly associated with WRC after Bonferroni correction (Figure 6). These results could be because the WRC data were not normally distributed or the phenotypic measurements recorded for WRC in this study are significantly influenced by nongenetic factors as noted by the low value of marker-based heritability. There were two markers located on the chromosome 4A that stood out from among the other SNPs: IWA4867 (562,407,344 bp) and IWA4698 (562,453,542 bp). Despite being not significant, these markers were examined further to determine if there may be any logical link to bran WRC. A majority of genotypes had these alleles for increased WRC: 192 genotypes for marker IWA4867 (TT genotype) and 194 for marker IWA4698 (TT genotype; Table 1; Supplementary Table S1). These genotypes were randomly distributed on a PCA biplot generated using the marker data, suggesting the absence of groupings among lines carrying the favorable alleles (Supplementary Figure S2).

As indicated, most genotypes carried the tentative alleles for increased bran WRC. Unfortunately, higher WRC of bran is unfavorable, because it is associated with production of bread with low loaf volume [9]. While the vast majority of breeding efforts have been focused on improving the performance of the refined flour, limited attention was dedicated to bran quality. Therefore, more efforts are needed to select lines for whole grain baking. Perhaps this can be done by selecting lines that do not have these alleles.

### 3.6. Candidate Genomic Regions for Bran Friability and WRC

The two markers that were significantly associated with bran friability, BS00000020_51 (5D: 3,609,894 bp) and Excalibur_c49805_63 (5D: 1,614,602 bp), were located in genes *TraesCS5D02G004300* (5D: 3,609,672–3,610,121 bp) and TraesCS5D02G001200 (5D: 1,609,486–1,614,664 bp). *TraesCS5D02G001200* encodes for sucrose membrane transfer proteins.As a part of cell membranes, these proteins could play an additional role in the friability of bran during milling. The other gene, *TraesCS5D02G004300*, was annotated as *Pinb-D1b* at Ensembl Plants [63] and as *Puroindoline-b* in the IWGSC functional annotations [62]. A BLASTN search with Pinb-D1 forward primer sequence (obtained from Graingenes; [66]) as query against the IWGSC RefSeq v1.0 wheat genome matched exactly to the first 20 bp of this gene. Further, the correlation coefficient between the SNP marker associated with bran friability (BS00000020_51) and *Pinb-D1b* marker (r = 0.75; *p* < 0.001) and *Pinb-Wild* (r = −0.70; *p* < 0.001) was relatively stronger compared to *Pina-D1* (r = 0.35; *p* = 0.02). This result is probably because the distance between *Pinb-D1* forward primer start position and the friability-associated SNP marker (BS00000020_51) is 222 bp. Overall, these observations indicate that the SNP marker associated with bran friability (BS00000020_51; 5D: 3 609 894 bp) is in linkage disequilibrium and potentially tagged to the Pinb-D1 marker, and is a functional variant for *Pinb* locus. This also suggests that *Pinb-D1* locus may be involved in regulating bran friability.

*Pinb-D1* genes encode for puroindoline proteins, which make up a complex multicomponent complex called fraibilin [67]. Friabilin is involved in the association between starch and protein in the wheat kernel and is responsible for soft endosperm texture [68]. The results from this study suggest that, in addition to controlling endosperm texture, the puroindoline genes may also affect bran texture.

The two SNP markers that were tentatively associated with WRC, IWA4867 (4A: 562,407,344 bp) and IWA4698 (4A: 562,453,542 bp), were located close to *TraesCS4A02G251100* (562,408,612–562,414,155 bp) and *TraesCS4A02G251300* (562,457,963–562,459,338 bp), respectively. The *TraesCS4A02G251300* gene did not have a characterized function in wheat. However, it did have high similarity to the *Zm00001d033902_T001* gene present in maize (%ID—89.5; E-value: 1.6 × 10^−63^), which was reported to encode for cysteine-rich secretory proteins. Interestingly, in wheat, nongluten cysteine-rich proteins are harmful for breadmaking because they interfere with gluten macropolymerization [69].

## 4. Conclusions

In conclusion, our study identified novel SNPs associated with bran friability and tentative SNPs associated with WRC. One of the SNP markers associated with bran friability was tagged to the well-characterized Pinb locus in wheat, which suggests that in addition to endosperm texture, Pinb may also have a role in bran texture (friability). The favorable alleles at the trait-associated SNP sites were present in only a fraction of lines (mostly recent releases and very few heritage or older cultivars) in the HWWAMP, which comprises breeding lines from many of the breeding programs in the US. This result indicates that the traits beneficial for whole wheat flour are neither selected for or against, just randomly, and are relatively new in the breeding history. Additionally, the lines carrying favorable alleles for both traits were randomly distributed in a PCA biplot, suggesting the absence of groupings among lines carrying favorable alleles. A conscious effort is thus needed to breed for cultivars that would be more desirable for whole-grain milling. Upon validation in independent populations and multiple environments, the novel markers identified in this study may help breeders select lines that have high bran friability and low WRC, which are preferred in whole grain bread-making. Additionally, the lines carrying favorable alleles identified in this study can be used as parental lines while making new crosses in breeding programs for developing cultivars suitable for whole wheat grain flour. Further, if the genomic regions for bran friability and WRC are validated in multiple backgrounds and environments, they can then become potential targets for function characterization studies such as knockout or overexpression experiments and thus help narrow down genes important for these traits in bread wheat. These efforts overall could lead to release of wheat lines with high whole grain flour functionality and identification and characterization of genes important for whole grain bread-making.

## Figures and Tables

**Table 1:** Trait-associated SNPs and their annotations.^a^

Trait	Marker	SNP	Chr.	Position (bp)	*p*-Value	Marker *R*^2^ ^b^	Additive Effect Score (Associated Genotype)	Genotype at SNP Site Associated with an Increase in the Phenotypic Value of the Trait (Number of Lines with This Genotype) ^a^	Genotype at SNP Site Associated with a Decrease in the Phenotypic Value of the Trait (Number of Lines with This Genotype)
Friability	BS00000020_51	A/G	5D	3,609,894	<0.001	0.12	−5.95 (AA)	GG (46)	AA (250)
	Excalibur_c49805_63	A/G	5D	1,614,602	<0.001	0.11	5.93 (AA)	AA (43)	GG (254)
WRC	IWA4867	C/T	4A	562,407,344	0.0002	0.05	−20.5 (CC)	TT (192)	CC (97)
	IWA4698	C/T	4A	562,453,542	0.0002	0.04	−19.9 (CC)	TT (194)	CC (98)

^a^ Out of a total of 297 lines analyzed from the Hard Winter Wheat Association Mapping Panel; ^b^ Proportion of phenotypic variance explained by the SNP marker.

## Supplementary Materials

The following are available online at https://www.mdpi.com/2073-4425/11/8/838/s1, Figure S1: Principle components analysis biplot of the 297 genotypes in the HWWAMP using marker data, with the 46 lines carrying the GG favorable genotype for the friability marker, BS00000020_51 (A), highlighted in color, and the 43 lines carrying the AA favorable genotype for the friability marker, Excalibur_c49805_63 (B), highlighted in color, Figure S2: Principle components analysis biplot of the 297 genotypes in the HWWAMP using marker data, with the 97 lines carrying CC favorable genotype for the WRC marker, IWA4867 (A), highlighted in color, and the 98 lines carrying CC favorable genotype for the WRC marker, IWA4698, highlighted in color, Table S1: Genotype information across HWWAMP lines for the markers associated with Friability and Water Retention Capacity traits.
