# Supplementary material for: Insights into the Genetic Architecture of Bran Friability and Water Retention Capacity, Two Important Traits for Whole Grain End-Use Quality in Winter Wheat
Source: Genes (Basel). 2020 Jul 23;11(8):838. doi: 10.3390/genes11080838 (PMC7466047; doi:10.3390/genes11080838)
Supplement: Supplementary file 1 [file genes-11-00838-s001.pdf]

Supplementary Table 1. Genotype information across HWWAMP lines for the markers associated with Friability and Water Retention Capacity traits

marker	BS00000020_51	Excalibur_c49805_63	IWA4867	IWA4698
alleles	A/G	G/A	T/C	T/C
chrom	5D	5D	4A	4A
pos	3609894	1614602	562407344	562453542
AKRON	AA	GG	CC	CC
CAMELOT	AA	GG	TT	TT
CO04W320	AA	AA	CC	CC
LINDON	AA	GG	CC	CC
MILLENNIUM	AA	GG	TT	TT
NUFRONTIER	AA	AA	TT	TT
OK_RISING	AA	GG	CC	CC
OK10119	AA	GG	TT	TT
OK1068002	AA	GG	TT	TT
OVERLAND	AA	GG	TT	TT
PETE	AA	GG	TT	TT
POSTROCK	AA	GG	TT	TT
GALLAGHER	GG	GG	CC	CC
CENTURA	AA	GG	TT	TT
REDLAND	AA	GG	TT	TT
TAM107-R7	AA	GG	CC	CC
TAM203	GG	AA	CC	CC
TAM400	AA	GG	CC	CC
TX86A6880	AA	GG	CC	CC
MT9513	AA	GG	TT	TT
TX05V7269	AA	GG	CC	CC
TX05A001188	AA	GG	TT	TT
BILLINGS	AA	GG	TT	TT
DANBY	AA	GG	CC	CC
E2041	GG	GG	TT	TT
DENALI	AA	GG	CC	CC
CO050337-2	AA	GG	CC	CC
BYRD	AA	GG	TT	TT
CO07W245	AA	GG	CC	CC
MCGILL	AA	GG	TT	TT
NE02558	GG	AA	CC	CC
NW03666	AA	GG	TT	TT
NE04490	GG	AA	TT	TT
NE05430	AA	GG	TT	TT
NE05496	AA	GG	CC	CC

NE05548	AA	GG	TT	TT
NE06545	AA	GG	TT	TT
NE06607	AA	GG	TT	TT
ROBIDOUX	AA	GG	TT	TT
NI06736	AA	GG	CC	CC
NI06737	AA	GG	CC	CC
NI07703	AA	GG	TT	TT
NI08707	AA	GG	TT	TT
NI08708	AA	GG	TT	TT
BRONZE	AA	GG	TT	TT
NELL	AA	GG	TT	TT
WINOKA	AA	GG	TT	TT
ENDURANCE	AA	GG	TT	TT
AGATE	AA	GG	TT	TT
CENTERFIELD	GG	AA	TT	TT
CENTURK78	AA	GG	TT	TT
COLT	AA	GG	TT	TT
SCOUT66	AA	GG	TT	TT
OK05303	AA	GG	TT	TT
OK05312	AA	GG	CC	CC
OK05134	GG	AA	NN	NN
OK05526	AA	GG	CC	CC
OK05830	AA	GG	TT	TT
OK04507	GG	AA	CC	CC
PRONGHORN	AA	GG	TT	TT
HALLAM	AA	GG	TT	TT
ROSE	AA	GG	TT	TT
OK05511	AA	GG	CC	CC
CRIMSON	AA	GG	TT	TT
TANDEM	AA	GG	TT	TT
GUYMON	AA	GG	TT	TT
DUSTER	GG	GG	TT	TT
OK05711W	AA	GG	TT	TT
OK02405	AA	GG	TT	TT
GARRISON	AA	AA	TT	TT
OK05723W	GG	AA	TT	TT
OK05122	AA	GG	CC	CC
SD05118	AA	GG	TT	TT
SD05W018	AA	GG	TT	TT
OK04505	GG	AA	TT	TT
OK04525	GG	GG	TT	TT

OK04415	GG	AA	TT	TT
OK04111	GG	AA	TT	TT
TURKEY_NEBSEL	AA	GG	TT	TT
CENTURY	AA	GG	CC	CC
CUSTER	AA	GG	TT	TT
CHEYENNE	AA	GG	TT	TT
WENDY	AA	GG	TT	TT
HARDING	AA	GG	TT	TT
TRIUMPH64	AA	GG	CC	CC
CHISHOLM	AA	GG	TT	TT
OK06318	AA	GG	TT	TT
OK06336	AA	GG	TT	TT
DARRELL	AA	GG	TT	TT
GENT	AA	GG	CC	CC
OK06210	AA	GG	CC	CC
OK06319	GG	AA	CC	CC
KHARKOF	GG	GG	CC	CC
CULVER	AA	GG	TT	TT
ANTELOPE	AA	AA	TT	TT
BUCKSKIN	AA	GG	TT	TT
NIOBRARA	AA	GG	TT	TT
DELIVER	AA	GG	TT	TT
OK_BULLET	GG	AA	CC	CC
INTRADA	AA	GG	TT	TT
OK101	AA	GG	TT	TT
LANCER	AA	GG	TT	TT
WICHITA	AA	GG	CC	CC
WARRIOR	AA	GG	TT	TT
MACE	AA	GG	TT	TT
HARRY	AA	GG	TT	TT
VISTA	AA	GG	TT	TT
JERRY	AA	GG	TT	TT
GOODSTREAK	AA	GG	TT	TT
ARLIN	GG	AA	TT	TT
GAGE	AA	GG	TT	TT
WINDSTAR	AA	GG	TT	TT
NE99495	AA	GG	TT	TT
RAWHIDE	AA	GG	TT	TT
JAGGER	GG	AA	CC	CC
BENNETT	AA	GG	TT	TT
LYMAN	AA	GG	TT	TT

SD01058	AA	GG	TT	TT
SD01237	AA	GG	CC	CC
WAHOO	AA	GG	CC	CC
WESLEY	AA	GG	TT	TT
ALICE	AA	GG	TT	TT
ARAPAHOE	AA	GG	TT	TT
NEKOTA	AA	GG	CC	CC
ANTON	AA	GG	TT	TT
HOMESTEAD	AA	GG	TT	TT
OK05204	GG	AA	CC	CC
INFINITY_CL	AA	GG	NN	NN
OK06114	AA	GG	TT	TT
SIOUXLAND	AA	GG	TT	TT
OK05108	GG	AA	TT	TT
RITA	AA	GG	TT	TT
G1878	AA	GG	CC	CC
HV9W03-1379R	GG	AA	TT	TT
HV9W03-1551WP	AA	GG	TT	TT
SPARTAN	AA	GG	TT	TT
HV906-865	AA	GG	CC	CC
PROWERS	AA	GG	CC	CC
HV9W03-1596R	AA	GG	TT	TT
HV9W05-1280R	AA	GG	CC	CC
HV9W06-504	GG	AA	CC	CC
SMOKYHILL	AA	GG	TT	TT
ABOVE	AA	GG	CC	CC
VONA	AA	GG	CC	CC
SANDY	AA	AA	TT	TT
HAIL	AA	GG	TT	TT
BOND_CL	AA	GG	CC	CC
PLATTE	AA	GG	CC	CC
CO03W043	AA	GG	TT	TT
CO03W054	AA	GG	CC	CC
CO04025	GG	GG	TT	TT
CO04393	AA	GG	TT	TT
CO04499	AA	GG	CC	CC
LAMAR	AA	GG	CC	CC
CARSON	AA	GG	TT	TT
AVALANCHE	AA	GG	CC	CC
HALT	AA	GG	CC	CC

HATCHER	AA	GG	CC	CC
PRAIRIE_RED	AA	GG	CC	CC
YUMAR	AA	GG	CC	CC
SHOCKER	AA	GG	TT	TT
CO03064	AA	GG	CC	CC
BILL_BROWN	GG	GG	CC	CC
RIPPER	AA	GG	TT	TT
TARKIO	GG	AA	CC	CC
YUMA	AA	GG	CC	CC
JULES	AA	GG	CC	CC
LOCKETT	AA	GG	NN	NN
TAM302	AA	GG	TT	TT
TAM303	AA	GG	CC	CC
CAPROCK	AA	GG	CC	CC
TAM200	GG	GG	TT	TT
TAM112	AA	GG	TT	TT
TAM401	GG	AA	TT	TT
TAMW-101	AA	GG	TT	TT
TX01A5936	AA	GG	TT	TT
TAM105	AA	GG	CC	CC
TX86A8072	AA	GG	CC	CC
TAM304	GG	AA	TT	TT
TAM202	AA	GG	TT	TT
STURDY	AA	GG	CC	CC
TX86A5606	AA	GG	CC	CC
TX03A0563	AA	GG	TT	TT
TX03A0148	AA	GG	TT	TT
TX02A0252	AA	GG	TT	TT
TX04V075080	GG	AA	CC	CC
TX04M410211	AA	GG	TT	TT
MIT	AA	GG	CC	CC
STURDY_2K	GG	AA	TT	TT
2180	GG	AA	TT	TT
TAM107	AA	GG	CC	CC
HG-9	AA	GG	TT	TT
TX96D1073	AA	GG	TT	TT
TX99A0153-1	AA	GG	TT	TT
TX04M410164	AA	GG	TT	TT
TAM111	AA	GG	CC	CC
TX00V1131	GG	AA	TT	TT
TX01M5009-28	AA	GG	TT	TT

TX04A001246	AA	GG	TT	TT
TAM110	AA	GG	CC	CC
TAM109	AA	GG	TT	TT
TX01V5134RC-3	GG	AA	TT	TT
TX99U8618	AA	GG	TT	TT
HUME	AA	GG	TT	TT
2174-05	GG	AA	TT	TT
CREST	AA	GG	CC	CC
ROSEBUD	AA	GG	TT	TT
DECADE	AA	GG	TT	TT
MT06103	AA	GG	CC	CC
TX06A001263	AA	GG	TT	TT
TX05A001822	AA	GG	TT	TT
TX06A001132	AA	GG	CC	CC
TX06A001281	AA	GG	TT	TT
TX05V7259	AA	GG	TT	TT
TX07A001279	AA	GG	CC	CC
TX07A001318	AA	GG	CC	CC
TX07A001420	GG	AA	TT	TT
TX06V7266	AA	GG	TT	TT
OK1067071	AA	GG	NN	NN
OK1067274	AA	GG	TT	TT
OK1068009	AA	GG	TT	TT
OK1068026	AA	GG	TT	TT
OK1068112	AA	GG	TT	TT
OK1070275	NN	GG	NN	NN
OK1070267	AA	GG	NN	TT
OK09634	AA	GG	TT	TT
OK07231	GG	AA	TT	TT
OK07S117	AA	GG	CC	CC
OK08328	AA	GG	NN	CC
TX06A001386	GG	AA	NN	TT
LAKIN	AA	GG	TT	TT
OVERLEY	AA	GG	CC	CC
BIG_SKY	AA	GG	CC	CC
MT0495	AA	GG	TT	TT
MT85200	AA	GG	TT	TT
ALLIANCE	AA	GG	TT	TT
MTS0531	AA	GG	CC	CC
FULLER	AA	GG	TT	TT
NUSKY	AA	GG	TT	TT

GENOU	AA	GG	CC	CC
COUGAR	AA	GG	TT	TT
YELLOWSTONE	AA	GG	TT	TT
MT9982	AA	GG	TT	TT
EXPEDITION	AA	GG	CC	CC
JUDEE	AA	GG	TT	TT
MT9904	AA	GG	TT	TT
NUPLAINS	AA	GG	TT	TT
OK102	AA	GG	CC	CC
NORRIS	AA	GG	CC	CC
DUKE	AA	GG	TT	TT
JUDITH	AA	GG	TT	TT
SD05210	GG	AA	TT	TT
NEWTON	AA	GG	TT	TT
LARNED	AA	GG	TT	TT
KAW61	AA	GG	CC	CC
STANTON	AA	GG	CC	CC
PARKER76	AA	GG	TT	TT
TASCOSA	AA	GG	TT	TT
TREGO	AA	GG	CC	CC
KIRWIN	AA	GG	TT	TT
BISON	AA	GG	TT	TT
KARL_92	AA	GG	TT	TT
SAGE	AA	GG	TT	TT
KIOWA	AA	GG	TT	TT
DODGE	AA	GG	TT	TT
TRISON	AA	GG	TT	TT
COMANCHE	AA	GG	TT	TT
NORKAN	AA	GG	TT	TT
EAGLE	AA	GG	TT	TT
CHENEY	AA	GG	TT	TT
SHAWNEE	AA	GG	TT	TT
HEYNE	AA	GG	CC	CC
2145	AA	GG	TT	TT
BAKERS_WHITE	GG	AA	TT	TT
BURCHETT	AA	GG	CC	CC
CO940610	AA	AA	CC	CC
COSSACK	AA	GG	TT	TT
CUTTER	GG	AA	CC	CC
DUMAS	AA	GG	TT	TT
ENHANCER	GG	GG	TT	TT

HONDO	AA	GG	TT	TT
JAGALENE	AA	GG	CC	CC
KEOTA	AA	GG	CC	CC
KS00F5-20-3	GG	AA	CC	CC
LONGHORN	AA	GG	TT	TT
NEOSHO	AA	GG	CC	CC
NUHORIZON	AA	GG	TT	TT
OGALLALA	AA	GG	TT	TT
ONAGA	AA	GG	CC	CC
RONL	AA	GG	CC	CC
SANTA_FE	AA	GG	CC	CC
SETTLER_CL	AA	GG	CC	CC
THUNDER_CL	GG	AA	TT	TT
THUNDERBOLT	AA	GG	TT	TT
VENANGO	AA	GG	TT	TT
W04-417	GG	AA	TT	TT
WB411W	GG	AA	TT	TT

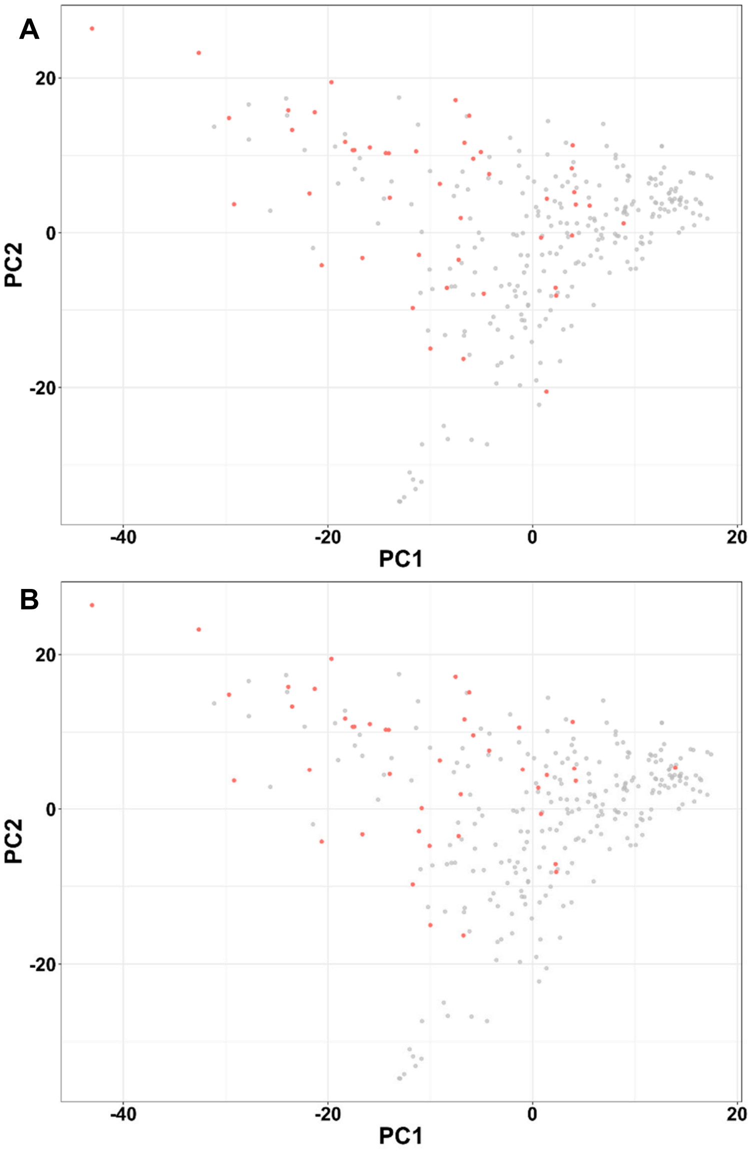

Supplementary Figure 1. Principle components analysis biplot of the 297 genotypes in the HWWAMP using marker data, with the 46 lines carrying the GG favorable genotype for the friability marker, BS00000020_51 (A), highlighted in color, and the 43 lines carrying the AA favorable genotype for the friability marker, Excalibur_c49805_63 (B), highlighted in color.

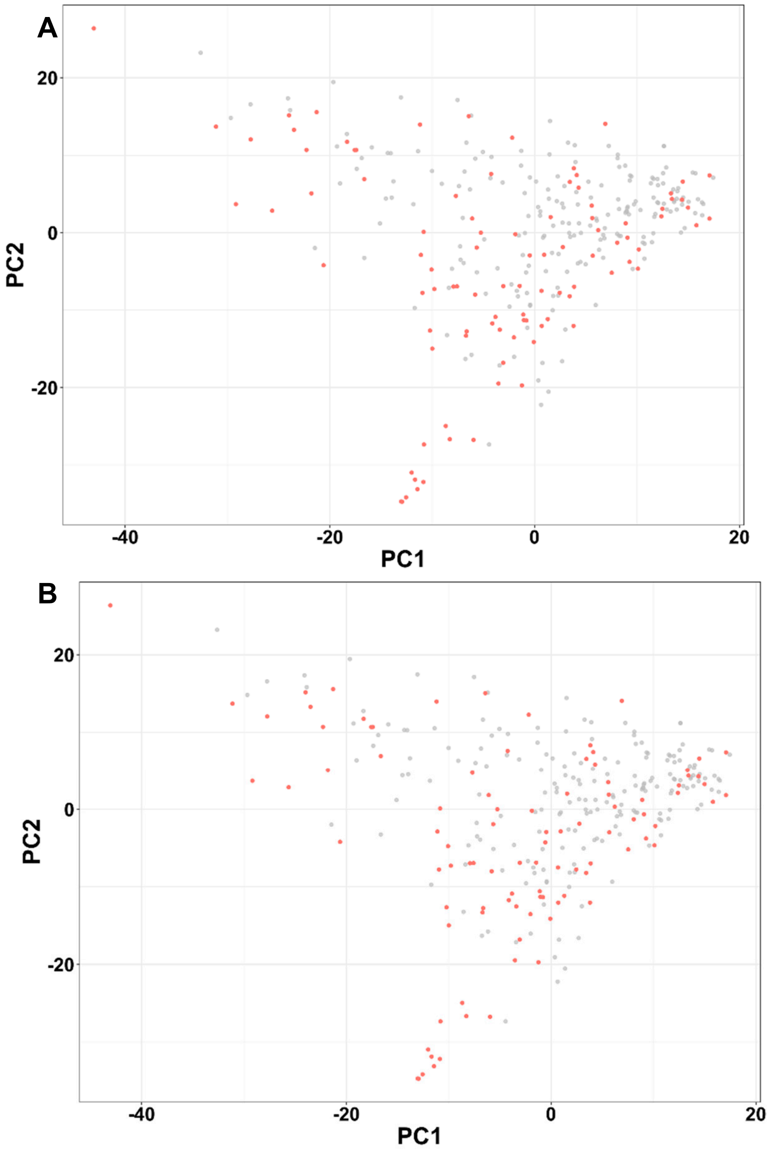

Supplementary Figure 2. Principle components analysis biplot of the 297 genotypes in the HWWAMP using marker data, with the 97 lines carrying CC favorable genotype for the WRC marker, IWA4867 (A), highlighted in color, and the 98 lines carrying CC favorable genotype for the WRC marker, IWA4698, highlighted in color.
